# Regulation of Non-coding RNAs in Heat Stress Responses of Plants

**DOI:** 10.3389/fpls.2016.01213

**Published:** 2016-08-18

**Authors:** Jianguo Zhao, Qingsong He, Gang Chen, Li Wang, Biao Jin

**Affiliations:** ^1^College of Horticulture and Plant Protection, Yangzhou UniversityYangzhou, China; ^2^College of Bio-Science and Bio-Technology, Yangzhou UniversityYangzhou, China; ^3^Key Laboratory of Crop Genetics and Physiology of Jiangsu ProvinceYangzhou, China

**Keywords:** ncRNA, heat stress, miRNA, siRNA, lncRNA

## Abstract

Heat stress is an important factor limiting plant growth, development, and productivity; thus, plants have evolved special adaptive mechanisms to cope with high-temperature stress. Non-coding RNAs (ncRNAs) are a class of regulatory RNAs that play an important role in many biological processes. Recently developed advanced technologies, such as genome-wide transcriptomic analysis, have revealed that abundant ncRNAs are expressed under heat stress. Although this area of research is still in its infancy, an increasing number of several classes of regulatory ncRNA (i.e., miRNA, siRNA, and lncRNA) related to heat stress responses have been reported. In this mini-review, we discuss our current understanding of the role of ncRNAs in heat stress responses in plants, especially miRNAs, siRNAs, and their targets. For example, the miR398-CSD/CCS-HSF, miR396-WRKY6, miR159-GAMYB, and TAS1-HTT-HSF pathways regulate plant heat tolerance. We highlight the hormone/development-related miRNAs involved in heat stress, and discuss the regulatory networks of miRNA-targets. We also note that DNA methylation and alternative splicing could affect miRNA expression under heat stress, and some lncRNAs could respond to heat stress. Finally, we briefly discuss future prospects concerning the ncRNA-related mechanisms of heat stress responses in plants.

## Introduction

Abiotic stresses, such as heat, drought, salinity, and low temperature, seriously impact the growth and productivity of plants. Consequently, as sessile organisms, plants have evolved various sophisticated mechanisms to cope with multiple abiotic stresses. In particular, given the increasing evidence of climate change, the heat stress response mechanism in plants has received increasing interest. Heat stress (high temperature) hinders cellular homeostasis and can lead to leaf etiolation, severe retardation in growth and development, increased risk of disease, and even death (Bita and Gerats, 2013; Liu et al., 2014). Most previous research focused on the regulatory mechanisms linking heat response stress to genes or transcription factors, such as the heat stress transcription factors (HSFs) regulating the expression of heat-responsive genes, and further affecting the accumulation of heat shock proteins (HSPs) in plant thermotolerance (Wang et al., 2004; Huang and Xu, 2008). Recently, however, more emerging non-coding RNAs (ncRNAs) have been found to play important roles in heat responses, the regulatory mechanisms of which were revealed in plants.

The ncRNAs constitute a class of RNA which does not encode a protein and includes microRNAs (miRNAs), small interfering RNAs (siRNAs), long non-coding RNAs (lncRNAs), and circular RNAs (circRNAs). These various types of ncRNA are involved in the transcriptional and post-transcriptional regulation of gene expression, and the modulation of RNA stability and translation (Hirayama and Shinozaki, 2010; de Lima et al., 2012; Khraiwesh et al., 2012). In recent years, a rapidly increasing number of ncRNAs have been reported to function in heat stress responses in plants (Table 1). In this mini-review, we summarize the plant ncRNAs involved in heat responses, focusing on miRNAs, siRNAs, and lncRNAs.

**Table 1:** **Non-coding RNAs responsive to heat stress in diverse plant species**.

**NcRNA**	**Species**	**Target**	**References**
miR156	Ath ↑ Tae ↑ Bra(h,g) ↑ Osa ↓	SPL	Xin et al., 2010; Yu et al., 2012; Sailaja et al., 2014; Stief et al., 2014
miR159	Tae ↑ Pvi ↑	MYB	Xin et al., 2010; Wang et al., 2012; Hivrale et al., 2016
miR160	Ath ↑ Hvu(a) ↑ Tae ↑ Pvi ↑ Han ↑ Agr ↑ Pto (a–c) ↓	ARF	Xin et al., 2010; Chen et al., 2012; May et al., 2013; Kruszka et al., 2014; Li M. Y. et al., 2014; Khaksefidi et al., 2015; Hivrale et al., 2016
miR164	Ath ↑ Agr ↑ Pvi ↑	NAC	May et al., 2013; Li M. Y. et al., 2014; Hivrale et al., 2016
miR166	Ath ↑ Hvu ↑ Tae ↑ Pvi ↑	HD-ZIPIII	Xin et al., 2010; May et al., 2013; Kruszka et al., 2014; Hivrale et al., 2016
miR167	Hvu(h) ↑ Pto(c–d) ↑ Tae ↑ Pvi ↑ Han ↑ Os ↓	ARF	Xin et al., 2010; Chen et al., 2012; Kruszka et al., 2014; Sailaja et al., 2014; Khaksefidi et al., 2015; Hivrale et al., 2016
miR168	Tae ↑ Agr ↑ Pvi ↑ Osa ↓ Pto(a–b) ↓	AGO1	Xin et al., 2010; Chen et al., 2012; Li M. Y. et al., 2014; Sailaja et al., 2014; Hivrale et al., 2016
miR169	Tae ↑ Ath ↑ Pto ↓	NF-Y	Xin et al., 2010; Chen et al., 2012; Guan et al., 2013
miR171	Ath ↑ Pto ↓ Pvi ↓ Ptc ↓	SCL	Lu et al., 2008; Chen et al., 2012; Mahale et al., 2013; Hivrale et al., 2016
miR172	Tae ↓ Ath ↓ Han ↓	AP2	Xin et al., 2010; May et al., 2013; Khaksefidi et al., 2015
miR319	Pvi ↑ Tae ↓	TCP	Kumar et al., 2014; Hivrale et al., 2016
miR390	Pvi ↑	ARF	Hivrale et al., 2016
miR393	Tae ↑ Pvi ↑ Ath ↓	TIR1/ AFB	Xin et al., 2010; Guan et al., 2013; Hivrale et al., 2016
miR394	Pto (a,b) ↓ Agr ↑	F-box	Chen et al., 2012; Li M. Y. et al., 2014
miR395	Tae ↑ Agr ↑ Pvi ↑ Pto (a–j) ↓	APS/AST	Chen et al., 2012; Kumar et al., 2014; Li M. Y. et al., 2014; Hivrale et al., 2016
miR396	Han ↑ Pvi ↑	GRF, bHLH, WRKY	Giacomelli et al., 2012; Hivrale et al., 2016
miR397	Ath(a) ↓ Osa(b) ↑	Laccases	Jeong et al., 2011; Mahale et al., 2013
miR398	Ath ↑ Han ↑ Tae ↑ Bra(a–b) ↓ Osa ↓ Pvi ↓ Pto(a-b) ↓	CSD, CCS, COX5	Xin et al., 2010; Chen et al., 2012; Yu et al., 2012; Guan et al., 2013; Lu et al., 2013; Sailaja et al., 2014; Khaksefidi et al., 2015; Hivrale et al., 2016
miR400	Ath ↓	PPR	Yan et al., 2012; Li S. X. et al., 2014
miR408	Agr ↑ Sja(b) ↓ Pto ↓ Pvi ↓	Plastocyanin	Chen et al., 2012; Li M. Y. et al., 2014; Liu et al., 2014; Hivrale et al., 2016
miR529	Pvi ↑	SBP-box	Hivrale et al., 2016
miR827	Tae ↑ Pvi ↑	SPX-MFS protein	Lin et al., 2010; Xin et al., 2010; Hivrale et al., 2016
miR5175	Hvu ↑	ACC-like oxidase	Kruszka et al., 2014
miR399	Tae ↑ Bra ↓	PHO2	Xin et al., 2010; Yu et al., 2012
SiRNA 002061_0636_3054.1	Tae ↓		Yao et al., 2010
SiRNA 005047_0654_1904.1	Tae ↓		Yao et al., 2010
SiRNA 080621_1340_98.1	Tae ↓		Yao et al., 2010
*TAS1*-siRNAs	Ath ↑	HTT1, HTT2	Li S. X. et al., 2014
lnRNA5	Tae ↑		Xin et al., 2011
lnRNA27	Tae ↑		Xin et al., 2011

Ath, Arabidopsis thaliana; Hvu, Hordeum vulgare; Ptc, Populus trichocarpa; Osa, Oryza sativa; Tae, Triticum aestivum; Pvi, Panicum virgatum; Han, Helianthus annuus; Agr, Apium graveolens; Pto, Populus tomentosa; Sja, Saccharina japonica; Bra, Brassica rapa; NF-Y, nuclear transcription factor Y; SBP, squamosa promoter binding; APS/AST, ATP sulfurylase/affinity sulfate transporter; TIR1/AFB, transport inhibitor response 1/auxin-related F-box; bHLH, basic-helix-loop-helix; GRF, growth hormone releasing factor; PHO2, phosphate 2; ↑, upregulated; ↓, downregulated.

## miRNAs

Plant miRNAs, a class of small (20–24 nucleotide) ncRNAs, negatively regulate gene expression by either mRNA degradation or translation inhibition (Rogers and Chen, 2013). Accumulating evidence has shown that miRNAs play essential roles in plant responses to heat stress (Table 1).

### miRNA398

**Figure 1:**
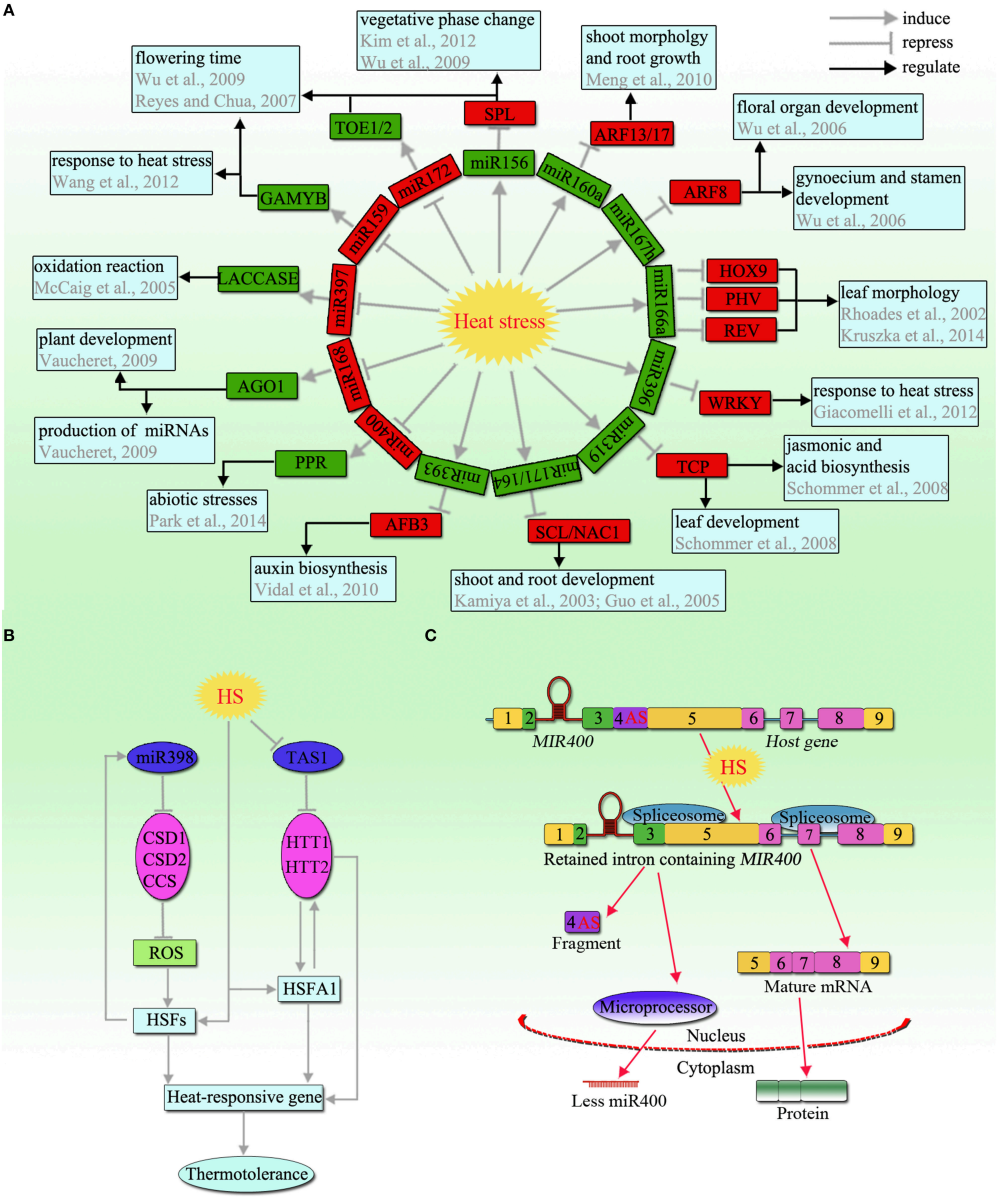
**An overview of non-coding RNAs in response to heat stress in plants. (A)** miRNA-target network module involved in the heat stress response. The network is based on the changes in expression profiles of miRNAs and their targets in plants under heat stress. Black arrows represent regulatory effects (position or negative regulation); Green boxes: upregulated; red boxes: downregulated. **(B)** Schematic model of miR398 and TAS1, which play an important role in thermotolerance. **(C)** Schematic model of the heat-induced AS that led to a decrease in miR400 expression (modified from Yan et al., 2012). Under heat stress, an alternative splicing (AS) event occurs in the miR400-containing intron and generates a new host gene. In addition, a fragment containing the original branch site is excised, which induces the rest of the unrecognized intron including the miR400 hairpin to be retained in the host gene. The primary miR400 transcripts without splicing out are hardly processed into mature miR400 by Microprocessor. The number 4 represents the AS intron region. Abbreviations: SPL, squamosa promoter binding protein-like; ARF, auxin response factor; HOX, homeobox leucine zipper protein; PHV, phavoluta; REV, revoluta; TCP, teosinte branched/cycloidea protein; SCL, scarecrow-like; NAC, nascent polypeptide-associated complex; AFB, auxin receptor F-box proteins; PPR, pentatricopeptide repeat; AGO, argonaute; GAMYB, gibberellic acid MYB; TOE, target of eat; CSD, copper/zinc superoxide dismutase; CCS, copper chaperone for superoxide dismutase; TAS1, trans-acting siRNA precursor 1; HTT, heat-induced tas1 target; ROS, reactive oxygen species; HSF, heat stress transcription factor; HS, heat stress.

miR398 is a specific well-studied example of an miRNA involved in responses to diverse abiotic stresses, particularly heat stress. In *Arabidopsis*, miR398 has four target genes, namely, *CSD1* and *CSD2* (closely related copper/zinc superoxide dismutases), *Cox5b-1* (a subunit of the mitochondrial cytochrome *c* oxidase), and *CCS1* (a copper chaperone for SOD; Sunkar and Zhu, 2004; Zhu et al., 2011), which are highly conserved in land plants. Among these, the CSDs are important scavengers of reactive oxygen species (ROS), and CSD/CCS negatively regulates the accumulation of ROS (Mittler, 2002; Sunkar et al., 2006), which are also associated with HSF and HSP synthesis (Guan et al., 2013; Lu et al., 2013). miR398 was shown to be rapidly induced in response to heat stress, accompanied by the downregulation of its target genes (CSD1, CSD2, and CCS; Guan et al., 2013; Figure 1B). Transgenic plants expressing miR398-resistant versions of *CSD1, CSD2*, or *CCS* showed hypersensitivity to heat stress, while the *csd1, csd2*, and *ccs* mutants were more tolerant to heat stress, with increased HSF and HSP levels (Guan et al., 2013; Lu et al., 2013). In addition, miR398 and its target *CSDs* were also found in the heat stress responses of *Brassica rapa* and *Populus tomentosa* (Kotak et al., 2007; Yu et al., 2012), indicating that the *miR398*-*CSD*/*CCS* pathway is widely involved in the heat stress response in plants.

### miR156 and miR172

miR156 and miR172 with their *SQUAMOSA PROMOTER BINDING PROTEIN-LIKE (SPL)* and *APETALA2* (*AP2*) targets control vegetative phase change and flowering (Wu et al., 2009). Under heat response conditions, miR156 was highly induced, and *SPL* was downregulated, which further induced *FLOWERINGLOCUS T* (*FT*) and *FRUITFULL* (*FUL*) expression in *Arabidopsis* (Kim et al., 2012; Stief et al., 2014). Similarly, miR156h and miR156g were particularly upregulated, and *BracSPL2* was sharply downregulated under heat stress in *B. rapa* (Yu et al., 2012). Interestingly, miR156 isoforms were important for heat stress memory in *Arabidopsis*. miR156 can promote the sustained expression of heat stress-responsive genes through *SPL* genes, especially *SPL2* and *SPL11*, and is critical only after heat stress (Cui et al., 2014; Stief et al., 2014). Owing to the conservation of miR156 and its target genes, it was proposed that the function of miR156 in heat stress memory may also be conserved in plants (Stief et al., 2014).

miR172 targets *AP2-like* genes, such as *TARGET OF EAT1* (*TOE1*), *TOE2*, and *SCHLAFMUTZE* (*SMZ*). In contrast to miR156, miR172 is downregulated by heat stress in plants (Figure 1A), such as in *Arabidopsis*, wheat, and *Helianthus annuus* (Table 1), while *TOE2* is upregulated (Li S. X. et al., 2014). Similarly, an elevated temperature also decreases miR172 expression and upregulates its target *TOE1* (May et al., 2013). The findings indicate that a high temperature can alter the expression of all components in the *miR156*-*SPL*-*miR172*-*AP2* pathway in a complex manner.

### Phytohormone-related miRNAs

A certain type of miRNA is associated with hormone responses to heat stress. Auxin signaling-related miR160 targets the *AUXIN RESPONSE FACTOR17* (*ARF17*) and *ARF13* genes, which are involved in root, shoot, and flower development (Meng et al., 2010). Under heat stress, miR160 was found to be upregulated while its target *ARF* was downregulated in *Hordeum vulgare* and *H. annuus* (Kruszka et al., 2014; Khaksefidi et al., 2015; Figure 1A). However, opposing patterns of miR160 regulation were reported in wheat, in which miR160 was downregulated while its other target *HSP70* was upregulated in response to heat stress (Kumar et al., 2014). Another auxin signaling-related miR167 that targets *ARF8*, which regulates floral organ and gynoecium and stamen development, was strongly decreased, while miR167h was increased significantly in response to heat stress (Wu et al., 2006; Kruszka et al., 2014; Figure 1A). In addition, many other miRNAs involved in the auxin signaling pathway, including miR390 and miR393, participate in the heat stress response (Vidal et al., 2010; Xin et al., 2010; Guan et al., 2013; Hivrale et al., 2016).

miR159 negatively regulates the gibberellic acid MYB (*GAMYB*) genes, which are important in seed germination and flower development (Reyes and Chua, 2007). In wheat, miR159 was downregulated with the upregulation of *TaGAMYB* after heat stress (Xin et al., 2010; Wang et al., 2012; Figure 1A). Rice mutants overexpressing *TamiR159* mutants or *Arabidopsis myb33myb65* double mutants (*TaGAMYB1* homologous genes) were heat-sensitive, indicating that the overexpression of miR159 led to *GAMYB* downregulation to decrease plant heat tolerance. In addition, miR319 [targets the teosinte branched/cycloidea proteins (TCPs) regulating jasmonic acid biosynthesis] was found to be upregulated and its targets *TCP2, TCP3*, and *TCP24* were downregulated under heat stress (Schommer et al., 2008; Li S. X. et al., 2014; Hivrale et al., 2016; Figure 1A). These findings indicate that many hormones related to miRNAs function in response to heat stress through miRNA-target gene networks.

### Development-related miRNAs

Heat stress significantly affects plant development, such as root and leaf development, seed germination, and photosynthesis. miR164 targets *nascent polypeptide-associated complex* (*NAC*) transcription factors to regulate shoot and root development (Guo et al., 2005). In *Arabidopsis*, miR164 was induced and *NAC1* was suppressed after high-temperature treatment (May et al., 2013; Li S. X. et al., 2014; Figure 1A). Particularly in wheat, miR164 could also directly target the heat shock protein *HSP17*, which showed upregulation under heat stress (Kumar et al., 2014). miR166 targets homeodomain-leucine zipper (HD-Zip) transcription factors regulating auxiliary meristem initiation and leaf morphology (Rhoades et al., 2002). In *H. vulgare*, miR166a was found to be upregulated while its targets *PHV* (*PHAVOLUTA*), *REV* (*REVOLUTA*), and *HOX9* (*homeobox leucine zipper protein HOX9-like*) were downregulated in response to heat (Kruszka et al., 2014; Figure 1A).

miR171 plays an important role in the expression of *SCARECROW-LIKE6- III* (*SCL6-III*) and *SCL6-IV* (GRAS family genes), which are known to be involved in many developmental processes, such as the radial patterning of both roots and shoots (Kamiya et al., 2003). In response to heat stress, miR171 was upregulated and further suppressed the expression of GRAS genes in *Arabidopsis* (Barku et al., 2013). However, different results were obtained in *Populus*, namely, pto-miR171 and ptc-miR171 were downregulated (Lu et al., 2008; Chen et al., 2012). *ARGONAUTE1* (*AGO1*) is one of the targets for miR168 and plays an important role in the production of miRNAs and in plant development (Vaucheret, 2009). miR168 was downregulated in response to heat, which would lead to a high level of *AGO1* (Chen et al., 2012; Figure 1A), suggesting that the miRNA-mediated regulation system is active under heat stress. Additionally, some other development-related miRNAs, including miR396 and its target *HaWRKY6*, and miR397 and its target *LACCASE*, were found to be associated with heat stress (McCaig et al., 2005; Giacomelli et al., 2012; Figure 1A), implying that miRNAs and their respective targets function in a complex regulatory network developed to cope with heat stress, and are involved in plant thermotolerance mechanisms.

### miRNA^*^

Mature miRNAs are excised as miRNA/miRNA^*^ duplexes from a precursor that resembles a hairpin structure. miRNA^*^ is a complementary strand of mature functional miRNA, and its expression level is very low compared with that of its miRNA counterpart. Previous investigations revealed the role of miRNA^*^ in plant stress responses. For example, miR393^*^ and miR399^*^ were upregulated in *Arabidopsis* by a bacterial pathogen and phosphate deprivation stress, respectively (Navarro et al., 2006; Zhang et al., 2011). Recent studies indicated that miRNA^*^ is also involved in the response to heat stress. For example, under heat stress, the expression of miRNA and miRNA^*^ variants of miR156h-2 was upregulated. In contrast, the miRNA^*^ variants of miR167a and miR400 were downregulated. In addition, the expression of both miR1885b.3 and miR1885b.3^*^ was severely suppressed by heat stress (Yu et al., 2012). A similar result was found for miR169^*^ and miR169 in switchgrass (*Panicum virgatum*) (Hivrale et al., 2016). Importantly, miR169^*^ has been predicted to target *bacterioferritin comigratory protein1* (*BCP1*) transcripts in *Medicago truncatula* (Devers et al., 2011), indicating that miRNA^*^ may regulate complementary mRNA targets (Zhang et al., 2011; Manavella et al., 2013). These findings show that miRNA^*^ is involved in heat response mechanisms in plants.

## Regulation of miRNA expression by alternative splicing

Alternative splicing (AS) is common in plants and contributes to both transcriptomic and proteomic diversity (Syed et al., 2012). Previous studies showed that many genes undergoing AS are involved in the regulation of plant responses to stress (Qin et al., 2007; Matsukura et al., 2010; Guerra et al., 2015). For example, the expression of *DEHYDRATION-RESPONSIVE ELEMENT BINDING 2B* (*DREB2B*) was shown to be regulated by AS in response to heat stress in *Zea mays* (Qin et al., 2007). In addition, heat stress-related AS was shown to regulate the expression level of miRNAs. For example, under heat stress, intronic miR400 was cotranscribed with its host gene and downregulated, while the expression level of miR400 primary transcripts was increased (Yan et al., 2012). This is interesting because an AS event was induced by heat stress, occurred in the intron where *MIR400* was located, and the heat stress-induced AS event inhibited mature miR400 expression (Figure 1C). Moreover, under heat stress, *Arabidopsis* seeds overexpressing miR400 had a lower germination rate (Yan et al., 2012). However, the miR400 target *pentatricopeptide repeat* (*PPR*), which is involved in plant development and abiotic stress, was upregulated by heat stress in *Arabidopsis* (Li S. X. et al., 2014; Park et al., 2014). In addition, some intronic miRNAs, including miR162a, miR788, miR838, miR844, miR848, miR853, and miR862, have potential AS isoforms, implying that through AS events, these intronic miRNAs may respond to stress in plants (Yan et al., 2012).

## DNA methylation-related miRNAs

DNA methylation is an important epigenetic modification, and plays a key role in the regulation of plant growth and development; it also has crucial functions in regulating gene expression in response to abiotic stress in plants (Rakei et al., 2015). For example, the expression of methylated *CycD3-1* and *Nt-EXPA5* was found to be altered during heat stress in tobacco (Centomani et al., 2015). In addition, under heat stress, DNA methylation might affect the expression of miRNAs and their targets (Ci et al., 2015). In *Populus simonii*, miR393a, miR156i, miR167h, miR396e, and miR396g genes were methylated at CNG sites in heat-treated plants, while they were methylated at CG sites in cold-treated ones. Under heat stress, miR390c with ^m^CG increased, while the expression of its target gene, *ISOCITRATE DEHYDROGENASE* (*IDH*), which participates in peroxisome biogenesis, was suppressed. Similarly, the expression of Ptc-miR156i and j with ^m^CNG modification was increased, but that of their target genes, *PHOSPHOLIPID*/*GLYCEROL ACYLTRANSFERASE FAMILY PROTEINS* (*LPCAT1* and *LPCAT2*), which are involved in ether lipid metabolism or glycerophospholipid metabolism, were suppressed under heat stress. In addition, miR396e/g were also induced, the targets of which are *ACYL-COA OXIDASES* (*ACOX1* and *ACOX3*), which function in alpha-linolenic acid metabolism and fatty acid degradation and were downregulated under heat stress (Biswas and Mano, 2015; Ci et al., 2015). From these results, under heat stress, DNA methylation might regulate miRNA expression, further affecting the expression level of their targets, likely through the gene-silencing function of miRNAs (Ci et al., 2015; Song et al., 2015).

## siRNAs

Small interfering RNAs (siRNAs) are approximately 21–24 nucleotide endogenous RNAs derived from the DCL family that catalyze the processing of double-stranded RNA (dsRNA) precursors (Axtell, 2013). According to their biogenesis and function, they can be further classified as *trans*-acting siRNAs (ta-siRNAs), natural antisense transcript siRNAs (nat-siRNAs), or heterochromatic siRNAs (Sunkar and Zhu, 2007; Axtell, 2013).

Sunkar and Zhu (2004) demonstrated that siRNAs are involved in abiotic stress responses in plants. Subsequently, a nat-siRNA, derived from natural *cis*-antisense transcript pairs of *SRO5* and *P5CDH* genes, was also found to regulate salt tolerance in *Arabidopsis* (Borsani et al., 2005). However, very little is known about the role of siRNAs in the heat stress response. In wheat seedlings, the expression levels of three siRNAs were downregulated by heat stress and upregulated by cold stress (Yao et al., 2010). *ONSEN*, a *copia*-type retrotransposon, was found to be activated in *Arabidopsis* seedlings under heat stress. In addition, the heat-induced accumulation of *ONSEN* was further significantly stimulated in mutants in which the biogenesis of siRNAs was impaired, indicating that siRNA-mediated regulation is responsible for the restriction of *ONSEN* transcript levels (Ito et al., 2011). Additionally, a high frequency of new *ONSEN* insertions was observed in the progeny of heat-stressed plants deficient in siRNAs (Matsunaga et al., 2012).

*Trans*-acting siRNAs (ta-siRNAs) are a specialized class of siRNAs that are generated by miRNA processing of a *TAS* gene transcript, the mode of action of which is very similar to that of miRNA (Axtell, 2013). The miR173-cleaved ta-siRNA (*TAS1*) targets *HEAT-INDUCED TAS1 TARGET1* (*HTT1*) and *HTT2* are involved in thermotolerance in *Arabidopsis*, and these targets were found to be highly induced by heat stress (Khraiwesh et al., 2012; Li S. X. et al., 2014; Figure 1B). The overexpression of *HTT1* and *HTT2* upregulated the accumulation of several *Hsf* genes to increase thermotolerance. Intriguingly, the *HTT* genes were also induced in mutants overexpressing *HsfA1a* under high temperatures. By contrast, the overexpression of *TAS1a* (*TAS1* family) caused higher sensitivity to heat stress through the elevated accumulation of *TAS1*-siRNAs and reduced expression levels of the *HTT* genes, suggesting that the *TAS1a* gene negatively regulates *HTT* and reduces thermotolerance.

Nat-siRNA is another siRNA whose dsRNA precursor is formed by the hybridization of two independently transcribed RNAs (Axtell, 2013). In *B. rapa*, differential expression analysis revealed that nat-siRNAs derived from 12 *cis*-NATs were responsive to heat stress, most of which showed strand bias. In addition, most of the transcripts generating heat-responsive nat-siRNAs were upregulated under heat stress, while the transcripts from the opposite strands of the same loci were downregulated (Yu et al., 2013).

## lncRNAs

lncRNAs in plants are more than 200 nt in length, distinguishing them from short ncRNAs (such as miRNA and siRNA). Based on their genomic locations, they are classified as antisense lncRNAs or intronic lncRNAs (Wierzbicki, 2012). Genome-wide scans have already revealed that lncRNAs are active in many plants (Zhang and Chen, 2013).

Despite limited reports on the mechanisms by which plant lncRNAs function, it was shown that they play vital roles in development and stress responses (Xin et al., 2011; Zhang and Chen, 2013). Several lncRNAs have been functionally characterized in plant stress-responsive pathways. For example, the lncRNAs *COOLAIR* (an antisense lncRNA) and *COLDAIR* (an intronic lncRNA) could be induced after vernalization to gradually suppress the expression of *FLC* (*FLOWERING LOCUS C*) (Heo and Sung, 2011). Under heat stress, lnc-173 was not induced, while its target gene *SUCROSE SYNTHASE 4* was responsive to a high temperature (Di et al., 2014). In wheat, Xin et al. (2011) characterized 125 putative long non-protein-coding RNAs (npcRNAs) during powdery mildew infection and heat stress, four of which were miRNA precursors (TalnRNA5, TalnRNA8, TalnRNA19, and TahlnRNA27). Among them, TalnRNA27 and TalnRNA5 were upregulated under heat stress. Di et al. (2014) identified 245 poly(A)+ and 58 poly(A)− lncRNAs that are differentially expressed under stress responses in *Arabidopsis*, and differential expression is significantly depleted in heat stress. Furthermore, 15 heat-responsive lncRNAs were validated by qRT-PCR. In *B. rapa*, under heat stress, 34 specifically expressed lncRNAs were identified, 192 target genes were regulated by lncRNAs and most of them belonged to the heat respond genes (Song et al., 2016). In addition, in *P. simonii*, the expression level of *PsiLncRNA00268512* was dynamic in response to heat stress (Song et al., 2015). Although some studies on the role of lncRNAs in plants have been performed, comprehensive surveys of lncRNA responses to heat stress are still lacking.

## Conclusions and perspectives

Plant ncRNAs play important roles in heat responses via ncRNA-target pathways comprising the heat stress response networks of plants. With the development of sequencing technologies and genome-scale approaches, ncRNAs and their targets responsive to heat stress are being extensively studied in organisms from model plant species to agricultural crops and non-agricultural species. Given that the majority of research in this field has involved identifying ncRNAs from different plant species, the pursuit of several worthwhile lines of study, such as functional analyses of specific ncRNAs, quantification of the effects of ncRNAs on their targets, ncRNA spatiotemporal-specific expression patterns, and even emerging circRNAs, should provide great insight into the complex ncRNA-mediated regulatory networks controlling plant heat response and tolerance.

## Author contributions

JZ and BJ wrote the manuscript. QH, GC, LW, and BJ reviewed and updated the manuscript.

### Conflict of interest statement

The authors declare that the research was conducted in the absence of any commercial or financial relationships that could be construed as a potential conflict of interest.

## References

[B1] Axtell M. J. (2013). Classification and comparison of small RNAs from plants. Annu. Rev. Plant Biol. 64, 137–159. 10.1146/annurev-arplant-050312-120043 23330790

[B2] Barku M. M. Bashasab F. Sudipta G. Krishnaraj P. U. (2013). LNA mediated *in situ* hybridization of miR171 and miR397a in leaf and ambient root tissues revealed expressional homogeneity in response to shoot heat shock in Arabidopsis thaliana. J. Plant Biochem. Biotechnol. 23, 93–103. 10.1007/s13562-013-0191-0

[B3] Biswas M. S. Mano J. I. (2015). Lipid peroxide-derived short-chain carbonyls mediate H_2_O_2_-induced and NaCl-induced programmed cell death in plants. Plant Physiol. 168, 885–898. 10.1104/pp.115.256834 26025050 PMC4741343

[B4] Bita C. E. Gerats T. (2013). Plant tolerance to high temperature in a changing environment: scientific fundamentals and production of heat stress-tolerant crops. Front. Plant Sci. 4:273. 10.3389/fpls.2013.00273 23914193 PMC3728475

[B5] Borsani O. Zhu J. Verslues P. E. Sunkar R. Zhu J. K. (2005). Endogenous siRNAs derived from a pair of natural cis-antisense transcripts regulate salt tolerance in *Arabidopsis*. Cell 123, 1279–1291. 10.1016/j.cell.2005.11.035 16377568 PMC3137516

[B6] Centomani I. Sgobba A. D'Addabbo P. Dipierro N. Paradiso A. De Gara L.. (2015). Involvement of DNA methylation in the control of cell growth during heat stress in tobacco BY-2 cells. Protoplasma 252, 1–9. 10.1007/s00709-015-0772-y 25712591

[B7] Chen L. Ren Y. Y. Zhang V. L. Xu V. L. Sun F. S. Zhang Z. Y.. (2012). Genome-wide identification and expression analysis of heat-responsive and novel microRNAs in Populus tomentosa. Gene 504, 160–165. 10.1016/j.gene.2012.05.034 22634103

[B8] Ci D. Song Y. P. Tian M. Zhang D. Q. (2015). Methylation of miRNA genes in the response to temperature stress in *Populus simonii*. Front. Plant Sci. 6:921. 10.3389/fpls.2015.00921 26579167 PMC4626561

[B9] Cui L. G. Shan J. X. Shi M. Gao J. P. Lin H. X. (2014). The *miR156*-*SPL9*-*DFR* pathway coordinates the relationship between development and abiotic stress tolerance in plants. Plant J. 80, 1108–1117. 10.1111/tpj.12712 25345491

[B10] de Lima J. C. Loss-Morais G. Margis R. (2012). microRNAs play critical roles during plant development and in response to abiotic stresses. Genet. Mol. Biol. 35(Suppl. 1), 1069–1077. 10.1590/s1415-47572012000600023 23412556 PMC3571433

[B11] Devers E. A. Branscheid A. May P. Krajinski F. (2011). Stars and symbiosis: microRNA-and microRNA-mediated transcript cleavage involved in arbuscular mycorrhizal symbiosis. Plant Physiol. 156, 1990–2010. 10.1104/pp.111.172627 21571671 PMC3149951

[B12] Di C. Yuan J. P. Wu Y. Li J. R. Lin H. X. Hu L.. (2014). Characterization of stress-responsive lncRNAs in *Arabidopsis thaliana* by integrating expression, epigenetic and structural features. Plant J. 80, 848–861. 10.1111/tpj.12679 25256571

[B13] Giacomelli J. I. Weigel D. Chan R. L. Manavella P. A. (2012). Role of recently evolved miRNA regulation of sunflower HaWRKY6 in response to temperature damage. New Phytol. 195, 766–773. 10.1111/j.1469-8137.2012.04259.x 22846054

[B14] Guan Q. M. Lu X. Y. Zeng H. T. Zhang Y. Y. Zhu J. H. (2013). Heat stress induction of miR398 triggers a regulatory loop that is critical for thermotolerance in *Arabidopsis*. Plant J. 74, 840–851. 10.1111/tpj.12169 23480361

[B15] Guerra D. Crosatti C. Khoshro H. H. Mastrangelo A. M. Mica E. Mazzucotelli E. (2015). Post-transcriptional and post-translational regulations of drought and heat response in plants: a spider's web of mechanisms. Front. Plant Sci. 6:57. 10.3389/fpls.2015.00057 25717333 PMC4324062

[B16] Guo H. S. Xie Q. Fei J. F. Chua N. H. (2005). MicroRNA directs mRNA cleavage of the transcription factor NAC1 to downregulate auxin signals for *Arabidopsis* lateral root development. Plant Cell 17, 1376–1386. 10.1105/tpc.105.030841 15829603 PMC1091761

[B17] Heo J. B. Sung S. (2011). Vernalization-mediated epigenetic silencing by a long intronic noncoding RNA. Science 331, 76–79. 10.1126/science.1197349 21127216

[B18] Hirayama T. Shinozaki K. (2010). Research on plant abiotic stress responses in the postgenome era: past, present and future. Plant J. 61, 1041–1052. 10.1111/j.1365-313X.2010.04124.x 20409277

[B19] Hivrale V. Yun Z. Puli C. O. R. Jagadeeswaran G. Gowdu K. Kakani G.. (2016). Characterization of drought- and heat-responsive microRNAs in switchgrass. Int. J. Plant Sci. 242, 214–223. 10.1016/j.plantsci.2015.07.018 26566839

[B20] Huang B. Xu C. P. (2008). Identification and characterization of proteins associated with plant tolerance to heat stress. J. Integr. Plant Biol. 50, 1230–1237. 10.1111/j.1744-7909.2008.00735.x 19017110

[B21] Ito H. Gaubert H. Bucher E. Mirouze M. Vaillant I. Paszkowski J. (2011). An siRNA pathway prevents transgenerational retrotransposition in plants subjected to stress. Nature 472, 115–119. 10.1038/nature09861 21399627

[B22] Jeong D. H. Park S. Zhai J. Z. Ranjan Gurazada S. G. Paoli E. D. Meyers B. C.. (2011). Massive analysis of rice small RNAs: mechanistic implications of regulated microRNAs and variants for differential target RNA cleavage. Plant Cell 23, 4185–4207. 10.1105/tpc.111.089045 22158467 PMC3269859

[B23] Kamiya N. Itoh J. Morikami A. Nagato Y. Matsuoka M. (2003). The SCARECROW gene's role in asymmetric cell divisions in rice plants. Plant J. 36, 45–54. 10.1046/j.1365-313X.2003.01856.x 12974810

[B24] Khaksefidi R. E. Mirlohi S. Khalaji F. Fakhari Z. Shiran B. Fallahi H.. (2015). Differential expression of seven conserved microRNAs in response to abiotic stress and their regulatory network in *Helianthus annuus*. Front. Plant Sci. 6:741. 10.3389/fpls.2015.00741 26442054 PMC4585256

[B25] Khraiwesh B. Zhu J. K. Zhu J. (2012). Role of miRNAs and siRNAs in biotic and abiotic stress responses of plants. BBA Gene Regul. Mech. 1819, 137–148. 10.1016/j.bbagrm.2011.05.001 21605713 PMC3175014

[B26] Kim J. J. Lee J. H. Kim W. Jung H. S. Huijser P. Ahn J. H.. (2012). The *miR156-SPL3* module regulates ambient temperature-responsive flowering via *FT* in *Arabidopsis thaliana*. Plant Physiol. 159, 461–478. 10.1104/pp.111.192369 22427344 PMC3375978

[B27] Kotak S. Larkindale J. Lee U. Koskull-Doring P. V. Vierling E. Scharf K. D. (2007). Complexity of the heat stress response in plants. Curr. Opin. Plant Biol. 10, 310–316. 10.1016/j.pbi.2007.04.011 17482504

[B28] Kruszka K. Pacak A. Swidabarteczka A. Nuc P. Alaba S. Wroblewska Z.. (2014). Transcriptionally and post-transcriptionally regulated microRNAs in heat stress response in barley. J. Exp. Bot. 65, 6123–6135. 10.1093/jxb/eru353 25183744 PMC4203144

[B29] Kumar R. R. Pathak H. Sharma S. K. Kale Y. K. Nirjal M. K. Singh G. P. (2014). Novel and conserved heat-responsive microRNAs in wheat (*Triticum aestivum* L.). Funct. Integr. Genomics 15, 1–26. 10.1007/s10142-014-0421-0 25480755

[B30] Li M. Y. Wang F. Xu Z. S. Jiang Q. Ma J. Tan G. F.. (2014). High throughput sequencing of two celery varieties small RNAs identifies microRNAs involved in temperature stress response. BMC Genomics 15:242. 10.1186/1471-2164-15-242 24673837 PMC3986682

[B31] Li S. X. Liu J. X. Liu Z. Y. Li X. R. Wu F. J. He Y. K. (2014). Heat-induced tas1 target1 mediates thermotolerance via heat stress transcription factor A1a–directed pathways in *Arabidopsis*. Plant Cell 26, 1764–1780. 10.1105/tpc.114.124883 24728648 PMC4036584

[B32] Lin S. I. Santi C. Jobet E. Lacut E. El K. N. Karlowski W. M.. (2010). Complex regulation of two target genes encoding spx-mfs proteins by rice miR827 in response to phosphate starvation. Plant Cell Physiol. 51, 2119–2131. 10.1093/pcp/pcq170 21062869

[B33] Liu F. L. Wang W. J. Sun X. T. Liang Z. R. Wang F. J. (2014). RNA-Seq revealed complex response to heat stress on transcriptomic level in Saccharina japonica (*Laminariales, Phaeophyta*). J. Appl. Phycol. 26, 1585–1596. 10.1007/s10811-013-0188-z

[B34] Lu S. F. Sun Y. H. Chiang V. L. (2008). Stress-responsive microRNAs in *Populus*. Plant J. 55, 131–151. 10.1111/j.1365-313X.2008.03497.x 18363789

[B35] Lu X. Guan Q. Zhu J. (2013). Downregulation of CSD2 by a heat-inducible miR398 is required for thermotolerance in *Arabidopsis*. Plant Signal. Behav. 8:e24952. 10.4161/psb.24952 23733060 PMC3999080

[B36] Mahale B. M. Fakrudin B. Ghosh S. Krishnaraj P. U. (2013). LNA mediated *in situ* hybridization of miR171 and miR397a in leaf and ambient root tissues revealed expressional homogeneity in response to shoot heat shock in *Arabidopsis thaliana*. J. Plant Biochem. Biotechnol. 23, 93–103. 10.1007/s13562-013-0191-0

[B37] Manavella P. A. Daniel K. Ignacio R. S. Burbano H. A. Claude B. Detlef W. (2013). Tissue-specific silencing of *Arabidopsis* SU(VAR) 3-9 HOMOLOG8 by miR171a. Plant Physiol. 161, 805–812. 10.1104/pp.112.207068 23204429 PMC3561020

[B38] Matsukura S. Mizoi J. Yoshida T. Todaka D. Ito Y. Maruyama K.. (2010). Comprehensive analysis of rice *DREB2*-type genes that encode transcription factors involved in the expression of abiotic stress-responsive genes. Mol. Genet. Genomics 283, 185–196. 10.1007/s00438-009-0506-y 20049613

[B39] Matsunaga W. Kobayashi A. Kato A. Ito H. (2012). The effects of heat induction and the siRNA biogenesis pathway on the transgenerational transposition of *ONSEN*, a *copia*-like retrotransposon in Arabidopsis thaliana. Plant Cell Physiol. 53, 824–833. 10.1093/pcp/pcr179 22173101

[B40] May P. Liao W. Wu Y. Shuai B. McCombie W. R. Zhang M. Q.. (2013). The effects of carbon dioxide and temperature on microRNA expression in *Arabidopsis* development. Nat. Commun. 4, 405–415. 10.1038/ncomms3145 23900278

[B41] McCaig B. C. Meagher R. B. Dean J. F. (2005). Gene structure and molecular analysis of the *laccase-like multicopper oxidase* (*LMCO*) gene family in *Arabidopsis thaliana*. Planta 221, 619–636. 10.1007/s00425-004-1472-6 15940465

[B42] Meng Y. Chen D. Ma X. Mao C. Cao J. Wu P.. (2010). Mechanisms of microRNA-mediated auxin signaling inferred from the rice mutant *osaxr*. Plant Signal. Behav. 5, 252–254. 10.4161/psb.5.3.10549 20023405 PMC2881269

[B43] Mittler R. (2002). Oxidative stress, antioxidants and stress tolerance. Trends Plant Sci. 7, 405–410. 10.1016/S1360-1385(02)02312-9 12234732

[B44] Navarro L. Dunoyer P. Jay F. Arnold B. Dharmasiri N. Estelle M.. (2006). A plant miRNA contributes to antibacterial resistance by repressing auxin signaling. Science 312, 436–439. 10.1126/science.1126088 16627744

[B45] Park Y. J. Lee H. J. Kwak K. J. Lee K. Hong S. W. Kang H. (2014). MicroRNA400-guided cleavage of pentatricopeptide repeat protein mRNAs renders *Arabidopsis thaliana* more susceptible to pathogenic bacteria and fungi. Plant Cell Physiol. 55, 1660–1668. 10.1093/pcp/pcu096 25008976

[B46] Qin F. Kakimoto M. Sakuma Y. Maruyama K. Osakabe Y. Tran L. S.. (2007). Regulation and functional analysis of ZmDREB2A in response to drought and heat stresses in Zea mays L. Plant J. 50, 54–69. 10.1111/j.1365-313X.2007.03034.x 17346263

[B47] Rakei A. Maali-Amiri R. Zeinali H. Ranjbar M. (2015). DNA methylation and physio-biochemical analysis of chickpea in response to cold stress. Protoplasma 253, 1–16. 10.1007/s00709-015-0788-3 25820678

[B48] Reyes J. L. Chua N. H. (2007). ABA induction of miR159 controls transcript levels of two MYB factors during *Arabidopsis* seed germination. Plant J. 49, 592–606. 10.1111/j.1365-313X.2006.02980.x 17217461

[B49] Rhoades M. W. Reinhart B. J. Lim L. P. Burge C. B. Bartel B. Bartel D. P. (2002). Prediction of plant microRNA targets. Cell 110, 513–520. 10.1016/S0092-8674(02)00863-2 12202040

[B50] Rogers K. Chen X. (2013). Biogenesis, turnover and mode of action of plant microRNAs. Plant Cell 25, 2383–2399. 10.1105/tpc.113.113159 23881412 PMC3753372

[B51] Sailaja B. Voleti S. R. Oleti D. Subrahmanyam N. Sarla V. Vishnuprasanth V. P. (2014). Prediction and expression analysis of miRNAs associated with heat stress in *Oryza sativa*. Rice Sci 21, 3–12. 10.1016/S1672-6308(13)60164-X

[B52] Schommer C. Palatnik J. F. Aggarwal P. Chételat A. Cubas P. Farmer E. E.. (2008). Control of jasmonate biosynthesis and senescence by miR319 targets. PLoS Biol. 6:e230. 10.1371/journal.pbio.0060230 18816164 PMC2553836

[B53] Song X. Liu G. Huang Z. Duan W. Tan H. Ying L.. (2016). Temperature expression patterns of genes and their coexpression with lncRNAs revealed by RNA-Seq in non-heading Chinese cabbage. BMC Genomics 17:297. 10.1186/s12864-016-2625-2 27103267 PMC4840866

[B54] Song Y. Ci D. Tian M. Zhang D. Q. (2015). Stable methylation of a non-coding RNA gene regulates gene expression in response to abiotic stress in *Populus simonii*. J. Exp. Bot. 67, 1477–1492. 10.1093/jxb/erv543 26712827

[B55] Stief A. Altmann S. Hoffmann K. Pant B. D. Scheible W. Bäurle I. (2014). *Arabidopsis* miR156 regulates tolerance to recurring environmental stress through *SPL* transcription factors. Plant Cell 26, 1792–1807. 10.1105/tpc.114.123851 24769482 PMC4036586

[B56] Sunkar R. Kapoor A. Zhu J. K. (2006). Posttranscriptional induction of two Cu/Zn superoxide dismutase genes in *Arabidopsis* is mediated by downregulation of miR398 and important for oxidative stress tolerance. Plant Cell 18, 2051–2065. 10.1105/tpc.106.041673 16861386 PMC1533975

[B57] Sunkar R. Zhu J. K. (2004). Novel and stress-regulated microRNAs and other small RNAs from *Arabidopsis*. Plant Cell 16, 2001–2019. 10.1105/tpc.104.022830 15258262 PMC519194

[B58] Sunkar R. Zhu J. K. (2007). Micro RNAs and short-interfering RNAs in plants. J. Integr. Plant Biol. 49, 817–826. 10.1111/j.1744-7909.2007.00499.x

[B59] Syed N. H. Kalyna M. Marquez Y. Barta A. Brown J. W. S. (2012). Alternative splicing in plants–coming of age. Trends Plant Sci. 17, 616–623. 10.1016/j.tplants.2012.06.001 22743067 PMC3466422

[B60] Vaucheret H. (2009). *AGO1* homeostasis involves differential production of 21-nt and 22-nt miR168 species by MIR168a and MIR168b. PLoS ONE 4:e6442. 10.1371/journal.pone.0006442 19649244 PMC2714465

[B61] Vidal E. A. Araus V. Lu C. Parry G. Green P. J. Coruzzi G. M.. (2010). Nitrate-responsive miR393/AFB3 regulatory module controls root system architecture in *Arabidopsis thaliana*. Proc. Natl. Acad. Sci. U.S.A. 107, 4477–4482. 10.1073/pnas.0909571107 20142497 PMC2840086

[B62] Wang W. X. Vinocur B. Shoseyov O. Altman A. (2004). Role of plant heat-shock proteins and molecular chaperones in the abiotic stress response. Trends Plant Sci. 9, 244–252. 10.1016/j.tplants.2004.03.006 15130550

[B63] Wang Y. Sun F. Cao H. Peng H. R. Ni Z. F. Sun Q. X.. (2012). TamiR159 directed wheat TaGAMYB cleavage and its involvement in anther development and heat response. PLoS ONE 7:e48445. 10.1371/journal.pone.0048445 23133634 PMC3486836

[B64] Wierzbicki A. T. (2012). The role of long non-coding RNA in transcriptional gene silencing. Curr. Opin. Plant Biol. 15, 517–522. 10.1016/j.pbi.2012.08.008 22960034

[B65] Wu G. Park M. Y. Conway S. R. Wang J. W. Weigel D. Poethig R. S. (2009). The sequential action of miR156 and miR172 regulates developmental timing in *Arabidopsis*. Cell 138, 750–759. 10.1016/j.cell.2009.06.031 19703400 PMC2732587

[B66] Wu M. F. Tian Q. Reed J. W. (2006). *Arabidopsis* microRNA167 controls patterns of ARF6 and ARF8 expression, and regulates both female and male reproduction. Development 133, 4211–4218. 10.1242/dev.02602 17021043

[B67] Xin M. Wang Y. Yao Y. Song N. Hu Z. Qin D.. (2011). Identification and characterization of wheat long non-protein coding RNAs responsive to powdery mildew infection and heat stress by using microarray analysis and SBS sequencing. BMC Plant Biol. 11:61. 10.1186/1471-2229-11-61 21473757 PMC3079642

[B68] Xin M. Yu W. Yao Y. Xie C. Peng H. Ni Z.. (2010). Diverse set of microRNAs are responsive to powdery mildew infection and heat stress in wheat (*Triticum aestivum* L.). BMC Plant Biol. 10:123. 10.1186/1471-2229-10-123 20573268 PMC3095282

[B69] Yan K. Liu P. Wu C. A. Yang G. D. Xu R. Guo Q. H.. (2012). Stress-induced alternative splicing provides a mechanism for the regulation of microRNA processing in *Arabidopsis thaliana*. Mol. Cell 48, 521–531. 10.1016/j.molcel.2012.08.032 23063528

[B70] Yao Y. Ni Z. Peng H. Sun F. Xin M. Sunkar R.. (2010). Non-coding small RNAs responsive to abiotic stress in wheat (*Triticum aestivum* L.). Funct. Integr. Genomics 10, 187–190. 10.1007/s10142-010-0163-6 20217168 PMC3059194

[B71] Yu X. Wang H. Lu Y. Z. Ruiter M. D. Cariaso M. Prins M.. (2012). Identification of conserved and novel microRNAs that are responsive to heat stress in *Brassica rapa*. J. Exp. Bot. 63, 1025–1038. 10.1093/jxb/err337 22025521 PMC3254694

[B72] Yu X. Yang J. Li X. Liu X. Sun C. Wu F.. (2013). Global analysis of cis-natural antisense transcripts and their heat-responsive nat-siRNAs in *Brassica rapa*. BMC Plant Biol. 13:208. 10.1186/1471-2229-13-208 24320882 PMC4029752

[B73] Zhang X. M. Zhao H. W. Gao S. Wang W. C. Surekha K. A. Huang H. D.. (2011). *Arabidopsis* argonaute 2 regulates innate immunity via miRNA393-mediated silencing of a golgi-localized *SNARE* gene, *MEMB12*. Mol. Cell 42, 356–366. 10.1016/j.molcel.2011.04.010 21549312 PMC3101262

[B74] Zhang Y. C. Chen Y. Q. (2013). Long noncoding RNAs: new regulators in plant development. Biochem. Biophys. Res. Commun. 436, 111–114. 10.1016/j.bbrc.2013.05.086 23726911

[B75] Zhu C. Ding Y. F. Liu H. L. (2011). MiR398 and plant stress responses. Physiol. Plant. 143, 1–9. 10.1111/j.1399-3054.2011.01477.x 21496029

